# Neurological and Other Papers

**Published:** 1920-09

**Authors:** 


### Reviews of Books.

**Neurological and other Papers.** Reprinted from the Writings of JOHN MICHELL CLARKE, M.A., M.D. Cantab., LL.D. Bristol, F.R.C.P. Lond., Lieut.-Col. R.A.M.C.(T.F.). Pp. xxiii., 263. Bristol: J. W. Arrowsmith Ltd. 1920. Price 10s. 6d. net.

To write a formal review of this volume would be an impossible task for anyone who had the privilege of working with the man in whose honour and commemoration it has been printed. Yet it is perhaps well to let his old friends know what kind of book this is by which his memory is to be perpetuated. For those who knew him intimately, and with the affection that was an inevitable outcome of such intimacy, it will be pleasant to find that the Editors, Drs. George Parker and J. M. Fortescue-Brickdale, to whom were entrusted the difficult task of preparing this volume, have done their work admirably. In a brief prefatory note they describe the foundation and collection of the Michell Clarke Memorial Fund, the decision of the Committee to apply part of that fund to the publishing of a volume of reprints, and the steps taken by them to prepare the volume. Then follows an excellent biographical memoir (reprinted from No. 135 of the *Journal*), which could not be bettered either in matter or manner; and after this a list of Michell Clarke's published contributions to the literature of medicine, numbering over a hundred, and classified according to the branch of medicine discussed. Approximately one-half fall under the heading of Neurology, the other half being devoted to diseases of the blood and ductless glands, acute infective diseases, and so on. From these the Editors have selected eight neurological papers and nine on general medicine, which are reprinted in the memorial volume. His old friends will be glad to know that among these is included Dr. Michell Clarke's Presidential Address to the Bristol Medico-Chirurgical Society on "The Relation of Medicine to Natural Science." Here, and perhaps in the opening paragraphs of his Long Fox Lecture (also included amongst the reprinted articles) there is a little unveiling of the inner mind of a man who was reticent about himself, even above his fellow-countrymen—a glimpse of the motives that actuated his tireless industry.

Those, again, who respected his work while knowing him less intimately, will find in the book a fair summary of his activities—work of the very first class in the domain of neurology, with contributions to the study of other branches of medicine that are scarcely less valuable. Much of his best work was published in forms that were unsuitable for reprint in this volume. Even so, however, we have here brought together into a convenient compass a number of papers that no student of internal medicine ought to ignore. In the Long Fox Lecture on "Cerebro-Spinal Syphilis" and in the Bradshaw Lecture on "The Nervous Affections of the Sixth and Seventh Decades of Life" there is an immense mass of valuable information set out with great care and skill. Indeed, it is not too much to say that every one of the papers included in the book is of value to all who are occupied in the study and practice of medicine. Moreover, this value will endure, because all that Dr. Michell Clarke wrote was derived from long and minute observation of clinical facts, interpreted by a mind that had built its superstructure of scientific medicine on a solid foundation of physiology and pathology.

A word must be said in recognition of the wisdom of those—the Editor of this *Journal* chief among them—who urged upon the Committee responsible for the expenditure of the Memorial Fund the desirability of preparing a volume of reprints such as we now have in our hands. The excellence of the editorial work has already been noticed. The publishers are also to be congratulated on producing so attractive a book in these difficult days. A good portrait of Dr. Michell Clarke forms a frontispiece.

The volume as a whole may be trusted to do what we feel sure he would wish: to prove that in this and other provincial schools of medicine in Britain the broadest and wisest views of our fight against disease are to be gained by those who will devote themselves to its study.

